# Evolving social contact patterns during the COVID-19 crisis in Luxembourg

**DOI:** 10.1371/journal.pone.0237128

**Published:** 2020-08-06

**Authors:** Ardashel Latsuzbaia, Malte Herold, Jean-Paul Bertemes, Joël Mossong

**Affiliations:** 1 Epidemiology and Microbial Genomics Unit, Laboratoire National de Santé, Dudelange, Luxembourg; 2 Luxembourg National Research Fund, Luxembourg, Luxembourg; Columbia University, UNITED STATES

## Abstract

We conducted an internet survey using Survey Monkey over six weeks to evaluate the impact of the government interventions on social contact patterns in Luxembourg. Participants were recruited via the science.lu website on March 25, April 2, April 16, May 1 during lockdown, and June 12 and June 25 after the lockdown to provide an estimate of their number of contacts within the previous 24 hours. During the lockdown, a total of 5,644 survey participants with a mean age of 44.2 years reported 18,118 contacts (mean = 3.2, IQR 1–4). The average number of contacts per day increased by 24% from 2.9 to 3.6 over the lockdown period. The average number of contacts decreased with age: 4.2 (IQR 2–5) for participants below 25 years and 1.7 (IQR 1–2) for participants above 64 years. Residents of Portuguese nationality reported a higher number of contacts (mean = 4.3, IQR 2–5) than Luxembourgish (mean = 3.5, IQR 2–4) or other foreign residents, respectively. After lockdown, 1,119 participants reported 7,974 contacts with 7.1 (IQR 3–9) contacts per day on average, of which 61.7% (4,917/7,974) occurred without a facemask (mean = 4.9, IQR 2–6). While the number of social contacts was substantially lower during the lockdown by more than 80% compared to the pre-pandemic period, we observed a more recent 121% increase during the post lockdown period showing an increased potential for COVID-19 spread. Monitoring social contacts is an important indicator to estimate the possible impact of government interventions on social contacts and the COVID-19 spread in the coming months.

## Introduction

**Fig 1:**
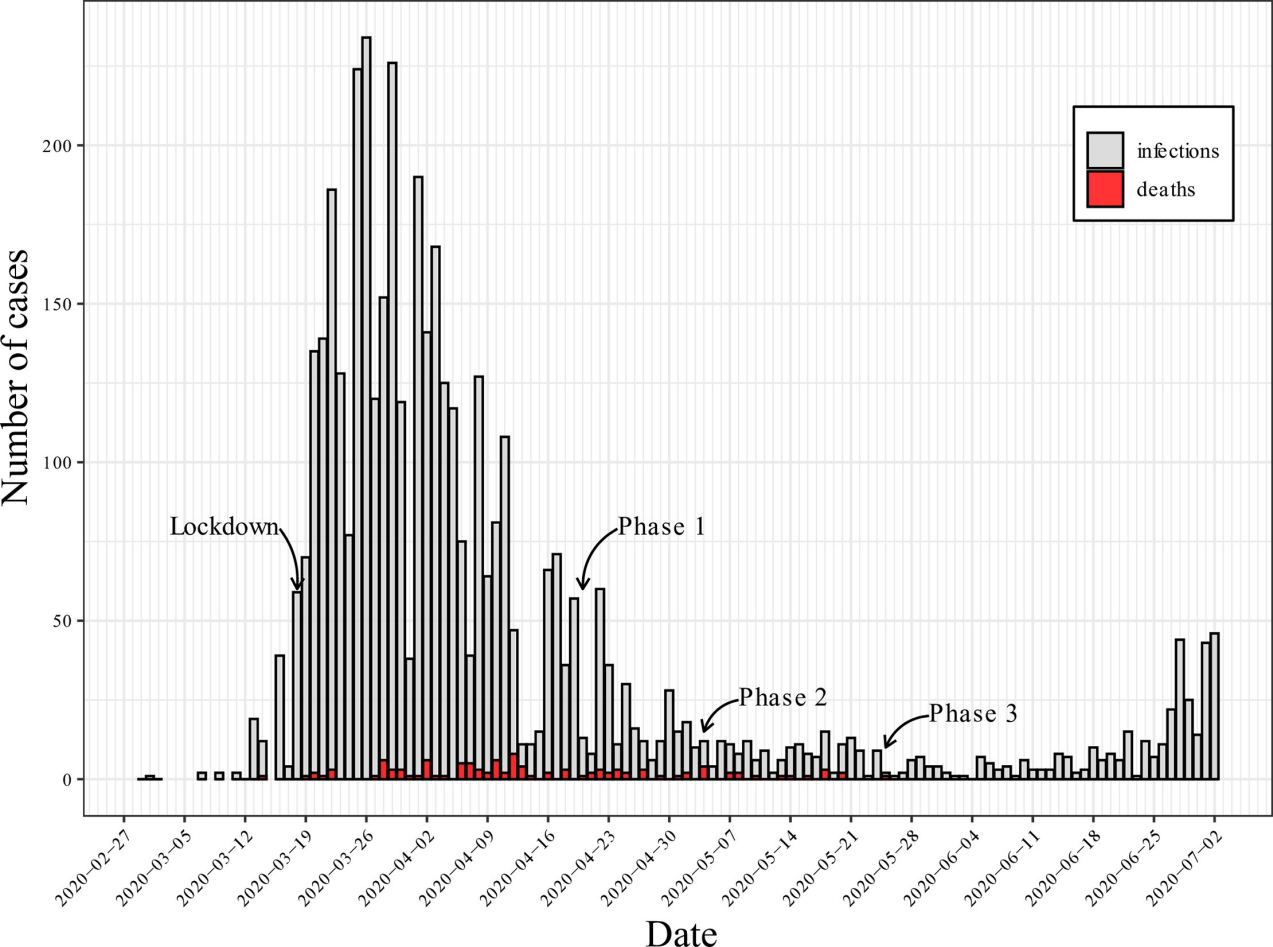
Incidence of confirmed cases and death.

COVID-19 has become a global public health emergency affecting more than 200 countries and territories resulting in more than 10 million reported cases by June 30 and over 500,000 deaths [1]. As of June 30, Luxembourg reported 4,299 cases and 110 deaths (Fig 1) [2]. Following the closure of schools, sports facilities, non-food shops, bars and restaurants on March 16, the Luxembourg government declared a state of emergency on March 18 implementing strict social distancing measures and instructing the local population to stay at home except for essential work and to avoid all unnecessary social interactions. Although social gatherings were prohibited, people were free to go outside while maintaining a physical distance of two meters or more. Five surgical masks were distributed to every resident on April 17–24 followed by the first easing of lockdown phase on April 20 with reopening of construction sites and recycling centres. The second phase started with reopening of final grades of secondary schools on May 4 and secondary schools one week later with reduced class size. Wearing a facemask became mandatory in a public area if a two-metre distance could not be maintained. Fifty additional surgical masks were distributed to every resident during the second easing of lockdown phase and gatherings of up to six people (plus household members) indoors and 20 people outdoors were authorised. The third phase was initiated on 25th of May reopening elementary schools with reduced class size, restaurants (maximum of 10 persons per table) and cafes (only table service) including mandatory face masks for staff and guests when not sitting at the table.

COVID-19 spreads via the respiratory route to close contacts and social contact patterns are therefore a key factor shaping the spread of COVID-19 and other infectious agents in a population [3, 4]. Contact surveys are an important methodological approach to assess social mixing as well the impact of control measures such as quarantine, travel restrictions or social distance measures, or lockdown in general [4–6]. Previous work from the UK suggests that lockdown measures may have decreased the reproduction number from 2.6 to 0.62 [6]. Similarly, researchers from China have shown a significant decrease of the reproduction number below one following physical restriction measures [7].

Social contact patterns differ across European countries. According to the POLYMED study Luxembourg resident reported 17.5 social contacts per day before the pandemic, similar to numbers reported for Italy (19.8). Belgian, British and German residents, for example, reported 11.8, 11.7 and 8.0 social contacts per day, respectively [4]. Additionally, Luxembourg has a unique demographic structure with a foreign population of nearly 50%, hence this population requires specific communication strategies targeting local and foreign communities.

We repeatedly conducted an internet survey to follow up the impact of the local government interventions on social contact patterns in Luxembourg shortly after the lockdown was implemented due to the rapid local spread of the COVID-19. In addition, our study provides insights on social contact patterns by age group and nationality, which can be important for identifying groups less compliant to imposed restrictions.

## Methods

### Study design and data collection

Recruitment of participants occurred via sharing of a survey link on the social media platforms Facebook and Twitter to followers and readers of the science.lu website following the publication of a general interest article on COVID-19 [8]. Individuals were requested to fill in an online questionnaire to self-report their daily number of contacts. The first survey was launched on the Survey Monkey platform on March 25, shortly after the state of emergency was declared (March 18) with a follow up survey launched on April 2, April 16 and May 1, respectively. For the post lockdown follow-up, survey participants were recruited on June 12 and 25. Each survey was open for participation for around 48 hours.

For the first wave, the survey collected age category, number of individuals living in the household other than the respondent and number of contacts within the last 24 hours excluding members of the household. From April 2, we expanded the questionnaire by recording nationality and the location where most contacts occurred (S1 File). The post lockdown survey included additional question to identify the number of contacts without wearing a facemask (S2 File). Two more categories were added to identify the place of contact with a multiple-choice answer.

A social contact was defined as a face-to-face conversation with more than three words at a distance of less than two meters. The total number of contacts was estimated by adding the reported number of contacts outside the household to the number of individuals living in the household.

Similarly, contacts without a facemask were calculated by adding the number of contacts without a facemask to individuals living in the household (assuming participants from the same household do not wear a mask at home).

The survey was purposely designed to have only a small number of questions (duration less than a minute) with available translations in three languages (German, French and English) to ensure high participation and completion.

Ethical approval for this study was waived by the Luxembourg Ministry of Health and the national ethics committee for research. Study participants were informed on how collected data was processed and utilized.

### Statistical analysis

The mean number of social contacts per person was calculated and stratified by age category, nationality, household size, location of most contacts and sampling week. The number of contacts in the questionnaire “10 or more” was counted as 10 contacts and “6–9” was averaged to 7.5. Similarly, in the post lockdown follow up survey, the number of contacts in the questionnaire “25 or more” was counted as 25 contacts. To estimate the number of contacts related to the place of occurrence, the total number of contacts was divided by the total number of places where contacts occurred assuming that participants would have a similar number of contacts in indicated places of contact.

We compared the mean number of daily participants to number of contacts from a large contact survey conducted in Luxembourg before the pandemic between May 2005 and September 2006 [4]. The total number of social contacts was adjusted representative to the population age structure (S1 Table). The age adjusted average number of reported contacts was calculated by multiplying the average number of contacts in each age group by the actual proportion of that age group from national population data [9]. A Poisson regression was performed to evaluate factors influencing the number of contacts. Variables significantly associated with the number of social contacts in univariate regression were selected for the multivariable model. The language variable was excluded from the model due to significant correlation with nationality (r = 0.45, p<0.05). The statistical analysis was performed in Stata 14 (College Station, Texas USA).

The effective basic reproduction number estimates and graph were downloaded from the EpiForecasts platform (https://epiforecasts.io/) [10, 11].

## Results

### Social contact patterns during lockdown

Between March 25 and May 1, a total of 5,644 (mean age 44.2 years) respondents participated in the online survey, of which 4.9% were under 25 years of age and 5.0% of participants were over 64 years of age (Table 1). Of 3,683 respondents reporting nationality, 60.3% (2,221/3,683) were Luxembourgish and 39.7% (1462/3,683) were foreign residents (Table 1).

**Table 1:** Number of reported contacts during lockdown.

Category	Covariate	N (%)	Mean number of contacts (IQR)	Pre-pandemic mean number of contacts (IQR)
Age category				
	13–17	44 (0.8)	4.2 (3, 4)	24.5 (14, 36)
	18–24	233 (4.1)	4.2 (2, 5)	19.4 (10, 26)
	25–34	977 (17.3)	3.1 (1, 4)	20.9 (12, 29)
	35–44	1,767 (31.3)	3.5 (2, 4)	19.2 (8, 28)
	45–54	1,381 (24.5)	3.6 (2, 4)	17.9 (8, 22)
	55–64	961 (17.0)	2.5 (1, 3)	12.5 (7, 16)
	65+	281 (5.0)	1.7 (1, 2)	8.7 (3, 13)
	Total	5,644 (100)	3.2 (1, 4)	17.5 (8, 24)
Household size				
	1	751 (13.3)	1.2 (0, 1)	13.1 (5, 16)
	2	1,492 (26.4)	2.1 (1, 2)	14.8 (7, 19)
	3	1,204 (21.3)	3.1 (2, 3)	16.9 (7, 24)
	4	1,261 (22.3)	4.1 (3, 4)	19.0 (9, 26)
	5	652 (11.6)	5.2 (4, 5)	18.4 (9, 25)
	≥6	287 (5.1)	6.4 (5, 7)	25.1 (23, 34)
Date				
	March 25	1,897 (33.6)	2.9 (1, 4)	-
	April 2	1,027 (18.2)	3.1 (1, 4)	-
	April 16	1,368 (24.2)	3.3 (1, 4)	-
	May 1	1,355 (24.0)	3.6 (2, 5)	-
Nationality				
	LU	2,221 (60.3)	3.5 (2, 4)	-
	FR	320 (8.7)	3.1 (1, 4)	-
	PT	195 (5.3)	4.3 (2, 5)	-
	BE	197 (5.4)	3.3 (1, 4)	-
	IT	121 (3.3)	3.1 (2, 4)	-
	DE	103 (2.8)	3.1 (1, 4)	-
	Others	526 (14.3)	2.9 (1, 4)	-
	Foreigners	1462 (39.7)	3.2 (1, 4)	-
Survey language				
	DE	2,004 (35.5)	3.2 (1, 4)	-
	FR	1,931(34.2)	3.6 (2, 5)	-
	EN	1,712 (30.3)	2.8 (1, 4)	-
Place of contact				
	supermarket	681 (27.8)	2.9 (1, 4)	-
	work	547 (22.3)	6.2 (3, 9)	24.6 (14, 32)
	leisure activity	330 (13.5)	3.2 (1, 4)	18.0 (9, 23)
	home	318 (13.0)	4.0 (2, 5)	11.6 (5, 15)
	other	572 (23.4)	3.7 (2, 4)	14.2 (7, 17)

Characteristics of study population for each wave are presented in S2 Table.

The total number of reported contacts was 18,118, while the average number of daily contacts was 3.2 (95% CI 3.1–3.3, IQR 1–4). After adjusting for age structure, the average number of daily contacts was 3.1. The average number of contacts reported by Luxembourg residents in a study before the pandemic was 17.5 [4], suggesting that contacts during lockdown had decreased by 81.7%. We observed a consistent decline across all age groups and household sizes.

The mean number of reported contacts was significantly higher (p<0.001) in young participants: 4.2 (95% CI 3.9–4.8, IQR 3–5) reported by participants below 25 years compared to 1.7 (95% CI 1.6–1.9, IQR 1–2) for participants above 64 years (Table 1).

Residents of Portuguese nationality reported a significantly higher (p<0.001) number of contacts (mean = 4.3 [95% CI 3.9–4.8, IQR 2–5]) than Luxembourgish residents (mean = 3.5 [95% CI 3.3–3.6, IQR 2–4] or other foreign residents (Table 1). The mean number of contacts was significantly higher (p<0.001) for the survey when conducted in French language (mean = 3.6 [95%CI 3.5–3.7, IQR 2–5]) compared to German (mean = 3.2 [95% CI 3.1–3.3, IQR 1–4]) and English (mean = 2.8 [95%CI 2.6–2.9, IQR 1–4]) language. We observed a significant variation of the average number contacts depending on the place where most contacts occurred. The highest number of contacts was reported for most contacts at work (mean = 6.2 [95% CI 5.9–6.5, IQR 3–9]), while the lowest number of contacts was reported for most contacts during leisure (mean = 3.2 [95%CI 3.0–3.5, IQR 1–4]) and at the supermarket (mean = 2.9 [95%CI 2.7–3.0, IQR 1–4]) (Table 1).

**Fig 2:**
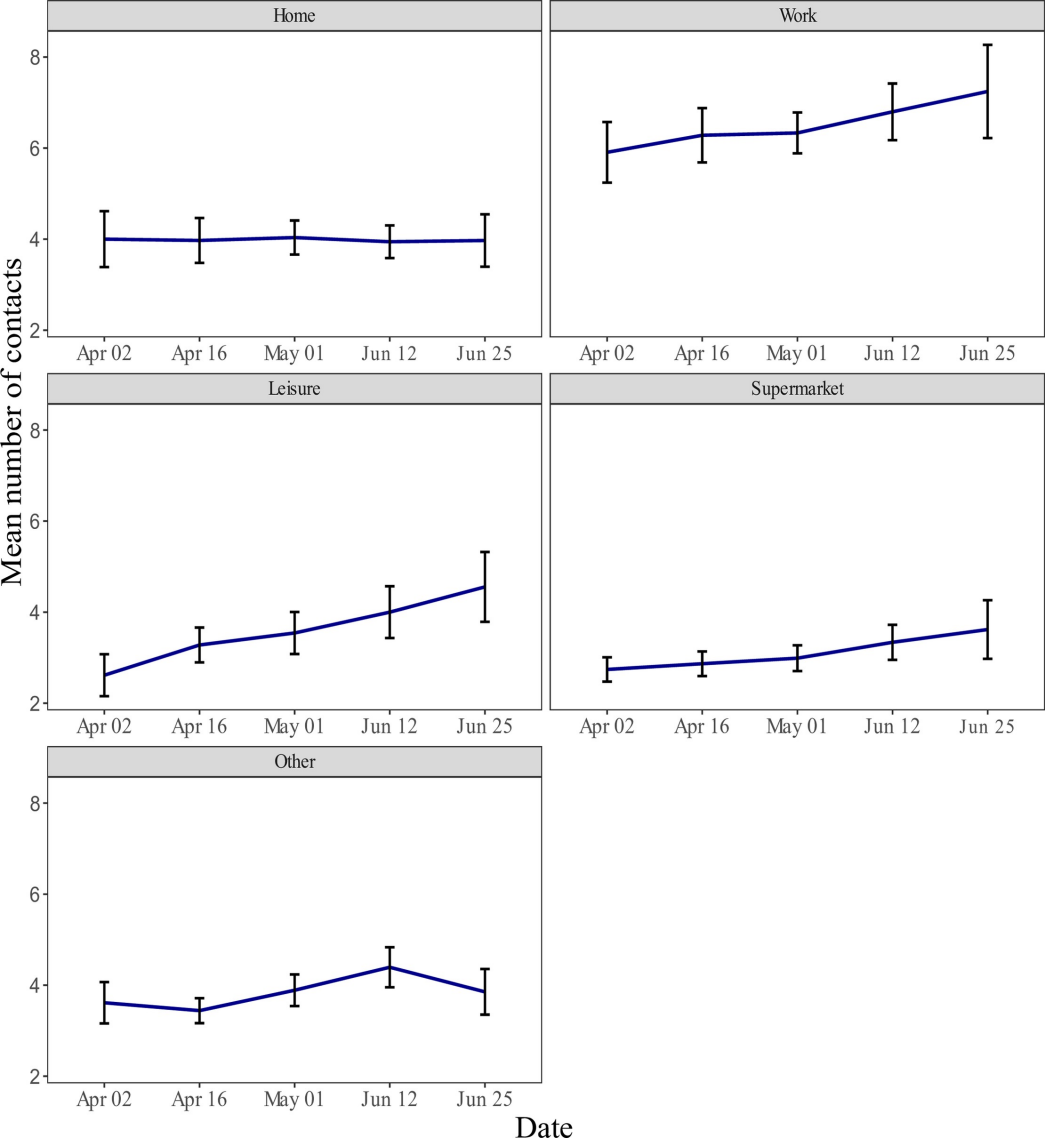
The average number of contacts stratified by date of survey^1^ and place of contact during lockdown (95%CI). ^1^ Place of contact was integrated in the survey from April 2^nd^.

The average number of contacts reported at work increased by 6.7% from 5.9 (95% CI 5.2–6.6, IQR 3–8) to 6.3 (95% CI 5.9–6.8, IQR 4–9), while average number of contacts during leisure activities increased by 34.6% from 2.6 (95% CI 2.2–3.1, IQR 1–4) to 3.5 (95% CI 3.5–4.0, IQR 2–5) (Table 1 and Fig 2).

**Fig 3:**
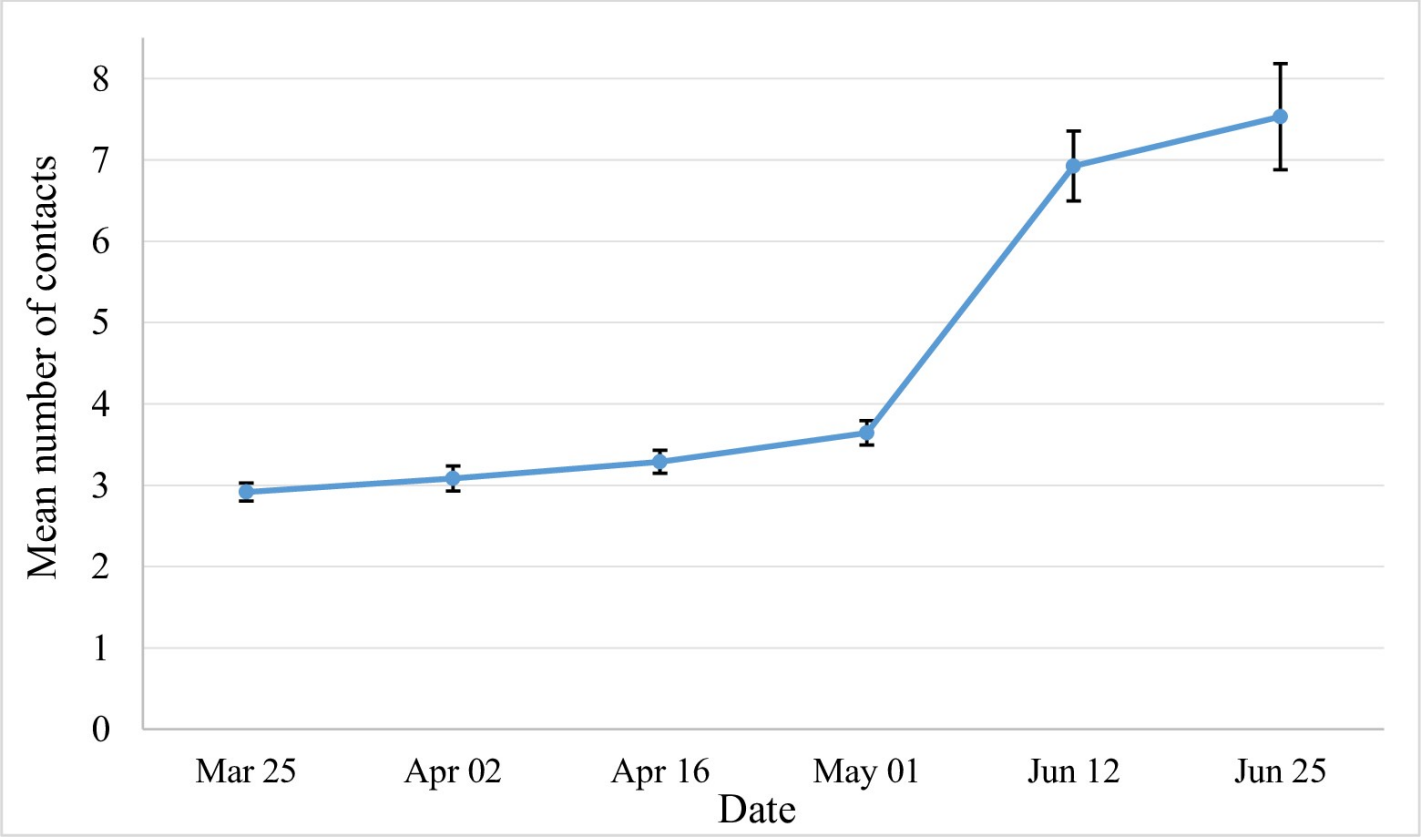
The average number of social contacts by date of survey (95%CI).

The average number of contacts per day significantly increased (p<0.001) by 24.1% over the lockdown period from 2.9 (95%CI 2.8–3.0, IQR 1–4) to 3.6 (95%CI 3.49–3.79, IQR 2–5) (Table 1 and Fig 3).

### Social contact patterns after lockdown

**Fig 4:**
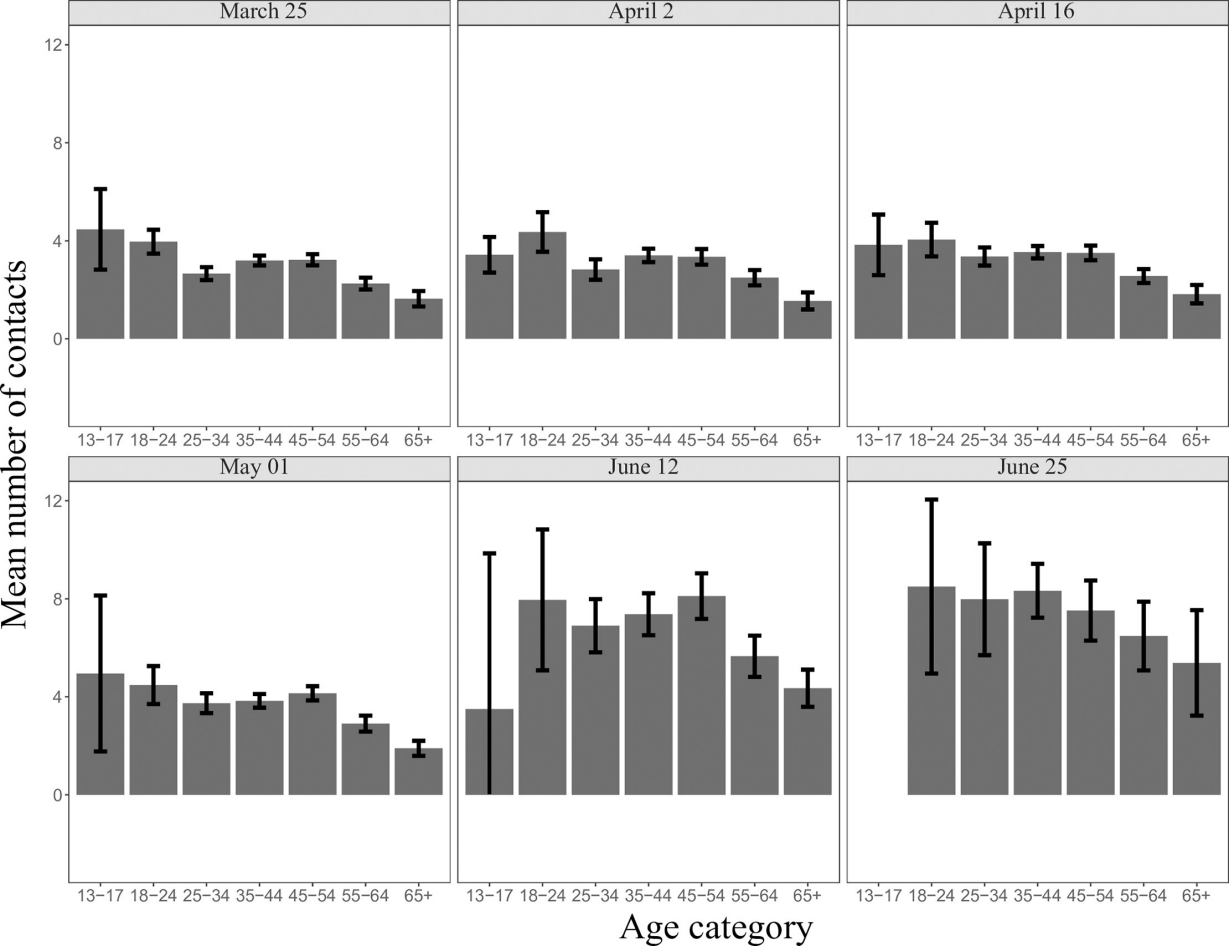
The average number of social contacts during and after lockdown stratified by age.

**Fig 5:**
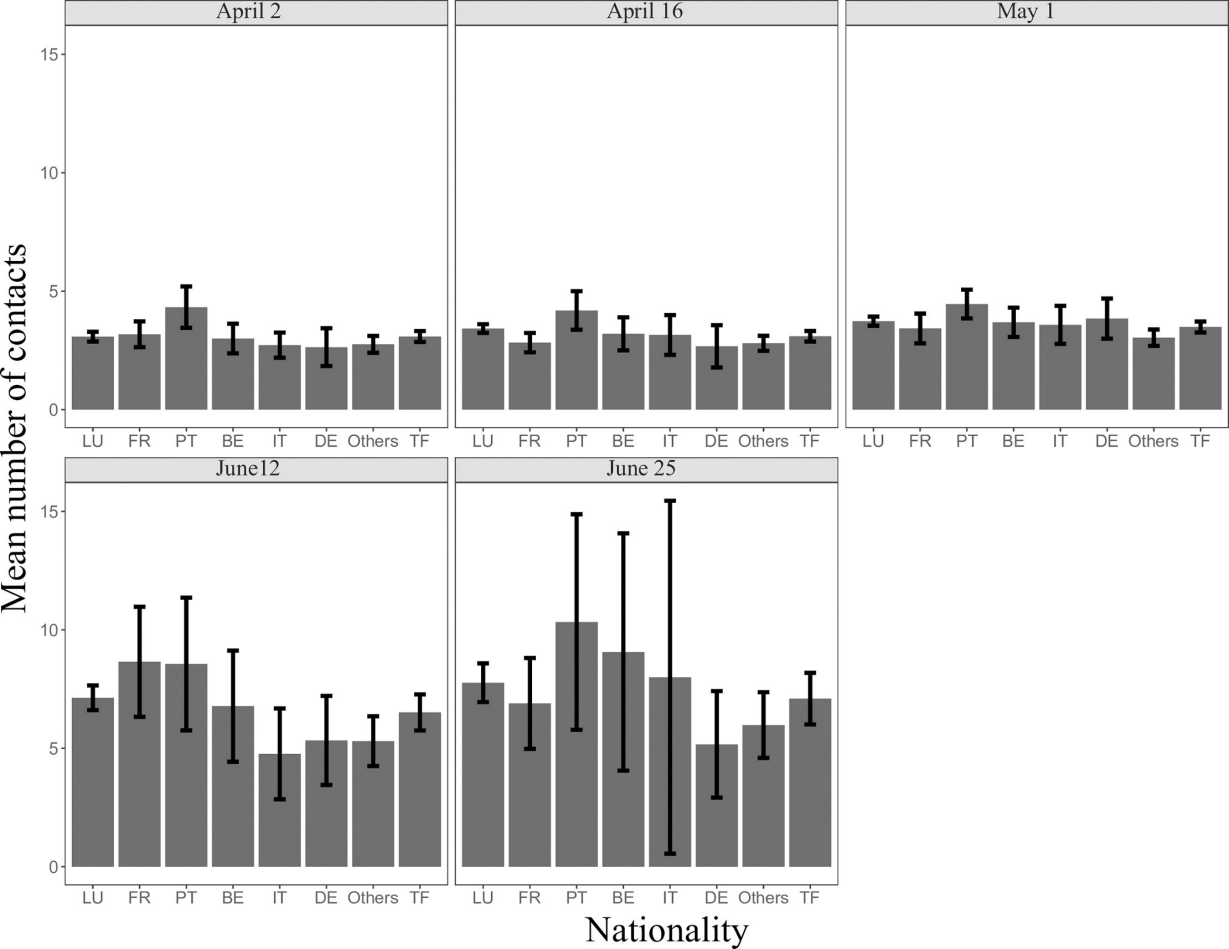
The average number of social contacts during and after lockdown stratified by nationality. LU, Luxembourgish; FR, French; PT, Portuguese; BE, Belgian; IT, Italian; DE, German; Others, other foreigners; TF, total foreigners.

In the post lockdown period, 1,119 participants filled in the survey (mean age = 46.3) reporting 7,974 contacts. The average number of daily contacts significantly increased from 3.2 during the lockdown to 7.1 after lockdown (95% CI 6.8–7.5, IQR 3–9) (Fig 3) (p<0.05). After adjusting for age structure, the average number of daily contacts was 6.7. The increase was consistent across all categories (Table 2, Figs 4 and 5). Of the total number of contacts, 61.7% (4,917/7,974) reported a contact without a facemask (mean = 4.9, IQR 2–6).

**Table 2:** Number of reported contacts after lockdown.

Category	Covariate	N (%)	Mean number of contacts after lockdown (IQR)	Mean number of contacts without facemask^1^
Age category				
	13–17	2 (0.2)	3.5 (3, 4)	3.5 (3, 4)
	18–24	40 (3.6)	8.2 (4, 9)	6.8 (4, 8)
	25–34	180 (16.1)	7.2 (3, 9)	4.8 (2, 6)
	35–44	304 (27.2)	7.7 (3, 11)	5.6 (3, 7)
	45–54	296 (26.5)	7.9 (4, 9)	5.0 (3, 6)
	55–64	206 (18.4)	5.9 (3, 7)	4.0 (2, 5)
	65+	89 (8.0)	4.7 (2, 6)	3.8 (2, 5)
	Total	1,117 (100)	7.1 (3, 9)	4.9 (2, 6)
Household size				
	1	162 (14.5)	4.3 (1, 6)	2.5 (1, 3)
	2	313 (28.0)	5.5 (2, 7)	3.5 (2, 4)
	3	248 (22.2)	7.3 (3, 8)	5.0 (2.6)
	4	225 (20.1)	8.7 (5, 11)	6.5 (4, 8)
	5	110 (9.8)	10.0 (6, 12)	7.5 (5, 8)
	≥6	61 (5.5)	11.7 (7, 14)	7.6 (5, 9)
Nationality				
	LU	497 (67.9)	7.1 (3, 9)	5.1 (1, 6)
	FR	52 (7.1)	8.6 (3, 11)	4.7 (2, 6)
	PT	25 (3.4)	8.6 (5, 13)	6.1 (3, 9)
	BE	27 (3.7)	6.8 (3, 7)	5.4 (3, 6)
	IT	17 (2.3)	4.8 (2, 7)	3.6 (1, 6)
	DE	15 (2.1)	5.3 (2, 8)	4.1 (2, 5)
	Others	99 (13.5)	5.3 (2, 7)	4.1 (2, 5)
	Foreigners	235 (32.1)	6.5 (2, 8)	4.6 (2, 6)
Survey language				
	DE	359 (47.9)	7.2 (3, 9)	5.1 (2, 6)
	FR	200 (26.7)	6.7 (4, 10)	5.4 (3, 6)
	EN	191 (25.5)	5.4 (2, 7)	4.1 (2, 5)
Place of contact				
	supermarket	199 (12.1)	3.4 (2, 4)	
	work	410 (25.0)	7.0 (3, 9)	
	leisure activity	202 (12.3)	4.2 (1, 5)	
	home	299 (18.2)	4.0 (2, 5)	
	restaurant/bar	166 (10.1)	4.0 (2, 5)	
	school	62 (3.8)	5.4 (3, 7)	
	other	295 (18.0)	4.2 (2, 6)	

^1^Including household members.

### Factors predicting the number of contacts

Univariate Poisson regression analysis showed that age above 55 years, foreign nationality (other than Portuguese), as well as English survey language were associated with a lower number of contacts (Table 3). Survey sampling week, Portuguese nationality and French survey language were associated with higher number of contacts. In multivariable regression, age, foreign nationality (other than Belgian) and calendar date remained significant predictors of the number of social contacts (Table 3).

**Table 3:** Poisson regression modeling total number of social contacts.

	Univariate regression			Multivariable regression		
Variable	RR	P-value	95% CI	RR	P-value	95% CI
Age category						
13–17	ref					
18–24	1.13	0.112	0.97–1.31	1.03	0.719	0.86–1.25
25–34	0.89	0.101	0.77–1.02	0.88	0.166	0.74–1.05
35–44	0.97	0.681	0.84–1.12	0.95	0.567	0.80–1.13
45–54	1.03	0.677	0.89–1.19	0.97	0.707	0.81–1.15
55–64	0.74	<0.001	0.64–0.86	0.70	<0.001	0.59–0.84
65+	0.58	<0.001	0.50–0.68	0.49	<0.001	1.41–0.59
Survey week						
March 25	ref					
April 2	1.05	0.013	1.01–1.10	0.42	<0.001	0.39–0.44
April 16	1.13	<0.001	1.08–1.17	0.43	<0.001	0.41–0.46
May 1	1.25	<0.001	1.20–1.30	0.49	<0.001	0.47–0.51
June 12	2.38	<0.001	2.29–2.47	0.95	0.022	0.90–0.99
June 25	2.58	<0.001	2.47–2.70	-		
Nationality						
Luxembourgish	ref					
French	0.92	0.003	0.88–0.97	0.92	0.001	0.87–97
Portuguese	1.18	<0.001	1.11–1.25	1.18	<0.001	1.11–1.25
Belgian	0.92	0.013	0.86–0.98	0.96	0.237	0.90–1.03
German	0.80	<0.001	0.73–0.88	0.82	<0.001	0.75–0.90
Italian	0.77	<0.001	0.70–0.84	0.83	<0.001	0.76–0.91
Others	0.76	<0.001	0.73–0.80	0.76	<0.001	0.73–0.79
Survey language						
German	Ref.					
English	0.77	<0.001	0.75–0.80	-	-	-
French	1.04	0.003	1.01–1.07	-	-	-

RR, rate ratio

Full model was adjusted by age, nationality and place of contact. Survey language is not included in the full model due to collinearity.

### Effective reproduction number of time

As shown in S1 Fig, the effective reproduction number in Luxembourg dropped below one shortly after lockdown (S1 Fig) [10, 11] and remained below one during the full lockdown period. The effective reproduction number increased to levels close to unity from the beginning of June onwards, although large confidence intervals were observed due to very low number of new daily cases.

## Discussion

Our study suggests that the strict physical distancing measures implemented in Luxembourg had a substantial and immediate impact on social mixing patterns resulting in a large reduction of the average number of contacts per day. During the early lockdown period, survey participants reported 3.2 contacts on average, which is 82% lower than during the non-pandemic period [4]. This decline was consistently observed across all age groups and household sizes. Our study findings are similar to those from Shanghai, Wuhan and the UK showing 88%, 86% and 73% reduction in the average number of daily contacts, respectively [6, 7]. In these studies, the reduction of contacts was estimated to have resulted in a significant decrease of the basic reproduction number R_0_ below one [5]. Similar to these estimates, our results explain the rapid decline in SARS-CoV-19 transmission, also resulting in the rapid decline of COVID-19 cases observed since the beginning of the lockdown in Luxembourg.

Although in Luxembourg the incidence of infections has been dropping to single figures by early May, further relaxing of physical distance restrictions poses significant risk for transmission. Relaxing restrictions too early could lead to an earlier second wave leading to further tightening of restrictions [12, 13].

Between March 25 and May 1, the average number of contacts increased by 19% associated with an increasing number of contacts at work and during leisure activities. This increase occurred after the easing of lockdown phase 1 on April 20, when construction sites and recycling centers were reopened. In June, during the post lockdown period the number of average contacts increased to 7.1, nevertheless it remains 59% lower than during the pre-pandemic period [4]. This increase in social contacts most likely resulted in the increase of the reproduction number followed by growing COVID-19 incidence that had been observed by the end of June. In addition, more than half of the contacts in the post lockdown period occurred without wearing a facemask increasing the transmission risk [14].

Our results suggest that older individuals are more compliant with restriction measures compared to younger persons, which is expected since the risk of hospitalization and death from COVID-19 increases with age [5]. Similarly, results of a large survey study conducted by Del Fava *et al*. showed that participants older than 65 years have a decreased number of contacts in Belgium, France, Germany, Italy, Netherlands, Spain and United Kingdom [15].

Residents of Portuguese nationality had more daily contacts compared to other residents, which could be work related, as 27% of Portuguese participants recorded contacts at work during lockdown. In Luxembourg, Portuguese residents represent the largest foreign community accounting for 15% of the total population and appear to be at increased risk for transmission [9]. Direct communication with these foreign communities could help to ensure compliance with physical distancing measures.

One limitation of our study is that we potentially overestimated the effect of lockdown due to selection bias. Compliant individuals following strict restrictions might be more active on social media and thus more likely to come across the survey. Secondly, we have to take into account that our study sample was not representative of the general population in terms of age structure and nationality: participants below 24 and above 65 years of age were underrepresented. Similarly, participants of Luxembourgish, French, Belgian and German nationality were overrepresented, Portuguese residents were underrepresented. Nevertheless, the adjusted mean number of contacts during lockdown was similar to the non-adjusted number of contacts. Another limitation of our study is that the pre-pandemic survey conducted in 2007 was conducted using a paper diary approach and our online approach might lead to lower ascertainment of contact numbers. Furthermore, the online survey does not account for multiple responses by a single respondent. We did not collect any further data on contacts (e.g. age), thus were unable to construct age matrix and estimate exact reduction of basic reproduction number. On the other hand, 6,766 respondents filled in the survey representing over 1% of total population in Luxembourg.

In conclusion, our stud shows that physical distance measures resulted in significant reduction in social contacts and therefore decreased the spread of COVID-19 in Luxembourg. Monitoring social contacts patterns using online surveys provides valuable early evidence of the effects of both lockdown and easying of lockdown measures on transmission and could be easily adapted to be used in different countries and regions in the world.

## Supporting information

S1 Fig Effective reproduction number estimates in Luxembourg. Time-varying estimate of the effective reproduction number. Light ribbon = 90% credible interval; dark ribbon = the 50% credible interval. Estimates are based on incidence data up to the 2020-06-22. Adopted from Abbott *et al*. estimating the time-varying reproduction number of SARS-CoV-2 using national and subnational case counts. (https://epiforecasts.io/covid/).
(PDF)

S1 Table Luxembourg population age structure and study population age structure. (DOCX)

S2 Table Characteristics of study participants by collection date. (DOCX)

S1 File (PDF)

S2 File (PDF)

S1 Raw data (CSV)
