# Supplementary figures and images for: Evolving social contact patterns during the COVID-19 crisis in Luxembourg
Source: PLoS One. 2020 Aug 6;15(8):e0237128. doi: 10.1371/journal.pone.0237128 (PMC7410209; doi:10.1371/journal.pone.0237128)

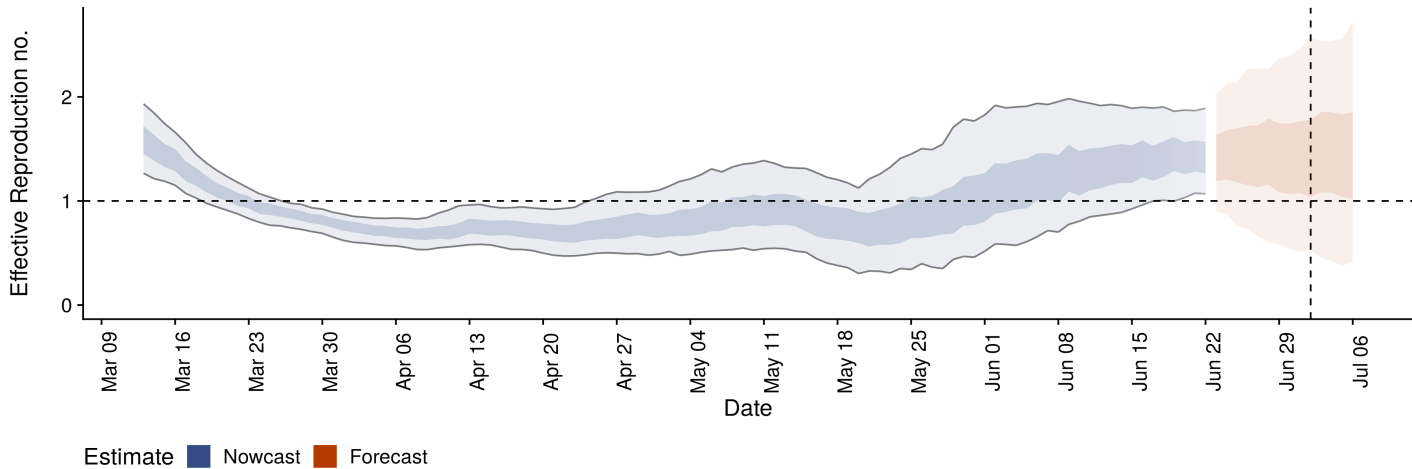

Supplement: S1 Fig — Time-varying estimate of the effective reproduction number. Light ribbon = 90% credible interval; dark ribbon = the 50% credible interval. Estimates are based on incidence data up to the 2020-06-22. Adopted from Abbott et al. estimating the time-varying reproduction number of SARS-CoV-2 using national and subnational case counts. (https://epiforecasts.io/covid/). (PDF) [file pone.0237128.s001.pdf]
