# Supplementary material for: Evolving social contact patterns during the COVID-19 crisis in Luxembourg
Source: PLoS One. 2020 Aug 6;15(8):e0237128. doi: 10.1371/journal.pone.0237128 (PMC7410209; doi:10.1371/journal.pone.0237128)
Supplement: S1 Table — (DOCX) [file pone.0237128.s002.docx]

S1 Table. Luxembourg population age structure and study population age structure

| Covariate | Luxembourg population | Study population during lockdown | Study population post-lockdown |
| --- | --- | --- | --- |
| Age group |  |  |  |
| 13-17 | 32,559 (6.0) | 44 (0.8) | 2 (0.7) |
| 18-24 | 52,409 (9.7) | 233 (4.1) | 40 (4.0) |
| 25-34 | 97,006 (18.0) | 977 (17.3) | 180 (17.1) |
| 35-44 | 96,209 (17.8) | 1,767 (31.3) | 304 (30.6) |
| 45-54 | 93,134 (17.3) | 1,381 (24.5) | 296 (24.8) |
| 55-64 | 77,024 (14.3) | 961 (17.0) | 206 (17.3) |
| 65+ | 90,787 (16.8) | 281 (5.0) | 89 (5.5) |
| 13-65+ | 539,128 (100.0) | 5,644 (100) | 1,117 (100) |
