# Supplementary material for: Evolving social contact patterns during the COVID-19 crisis in Luxembourg
Source: PLoS One. 2020 Aug 6;15(8):e0237128. doi: 10.1371/journal.pone.0237128 (PMC7410209; doi:10.1371/journal.pone.0237128)
Supplement: S2 Table — (DOCX) [file pone.0237128.s003.docx]

S2 Table. Characteristics of study participants by collection date.

| Category | Covariate | March 25  N (%) | April 2  N (%) | April 16  N (%) | May 1  N (%) | June 11  N (%) | June 25 | Total  N (%) |
| --- | --- | --- | --- | --- | --- | --- | --- | --- |
|  | 13-17 | 15 (0.8) | 7 (0.7) | 12 (0.9) | 10 (0.7) | 2 (0.3) | 0 (0) | 46 (0.7) |
|  | 18-24 | 81 (4.3) | 39 (3.8) | 63 (4.6) | 50 (3.7) | 22 (2.9) | 18 (4.9) | 273 (4.0) |
|  | 25-34 | 377 (19.9) | 152 (14.8) | 254 (18.6) | 194 (14.3) | 124 (16.6) | 57 (15.5) | 1,157 (17.1) |
|  | 35-44 | 605 (31.9) | 342 (33.3) | 429 (31.4) | 391 (28.9) | 197 (26.3) | 107 (29.0) | 2,071 (30.6) |
|  | 45-54 | 431 (22.8) | 246 (24.0) | 321 (23.5) | 383 (28.3) | 202 (45.5) | 94 (25.5) | 1,677 (24.8) |
|  | 55-64 | 296 (15.6) | 195 (19.0) | 239 (17.5) | 231 (17.1) | 139 (16.6) | 67 (18.2) | 1,167 (17.3) |
|  | 65+ | 89 (4.7) | 46 (4.5) | 50 (3.7) | 96 (7.1) | 63 (8.4) | 26 (7.1) | 370 (5.5) |
|  | Total | 1,894 (28.0) | 1,027 (15.2) | 1,368 (20.1) | 1,355 (20.0) | 749 (11.1) | 369 (5.5) | 6,761 (100.0) |
| Household size | | |  |  |  |  |  |  |
|  | 1 | 291 (15.3) | 118 (11.5) | 174 (12.7) | 168 (12.4) | 101 (13.5) | 61 (16.5) | 852 (13.3) |
|  | 2 | 521 (27.5) | 284 (27.7) | 341 (24.9) | 346 (25.5) | 211 (28.2) | 102 (27.6) | 1,703 (26.6) |
|  | 3 | 377 (19.9) | 213 (20.7) | 314 (23.0) | 300 (22.1) | 173 (23.1) | 75 (20.3) | 1,377 (21.5) |
|  | 4 | 407 (21.5) | 234 (22.8) | 306 (22.4) | 314 (23.2) | 156 (20.8) | 69 (18.7) | 1,417 (22.2) |
|  | 5 | 210 (11.1) | 128 (12.5) | 159 (11.6) | 155 (11.4) | 71 (9.5) | 39 (10.5) | 723 (11.3) |
|  | ≥6 | 91 (4.8) | 50 (5.0) | 74 (5.4) | 72 (5.0) | 37 (5.0) | 24 (6.5) | 324 (5.1) |
| Nationality |  |  |  |  |  |  |  |  |
|  | LU | - | 542 (53.8) | 811 (60.2) | 868 (65.4) | 497 (66.3) | 239 (64.6) | 2,718 (61.3) |
|  | FR | - | 101 (10.0) | 132 (9.8) | 87 (6.6) | 52 (6.9) | 28 (7.6) | 372 (8.4) |
|  | PT | - | 63 (6.3) | 75 (5.6) | 57 (4.3) | 25 (3.3) | 18 (4.9) | 220 (5.0) |
|  | BE | - | 52 (5.2) | 73 (5.4) | 72 (5.4) | 27 (3.6) | 15 (4.1) | 224 (5.1) |
|  | IT | - | 43 (4.3) | 46 (3.4) | 32 (2.4) | 17 (2.3) | 3 (0.8) | 138 (3.1) |
|  | DE | - | 33 (3.3) | 32 (2.4) | 38 (2.9) | 15 (2.0) | 12 (3.2) | 118 (2.7) |
|  | Others | - | 173 (17.2) | 179 (13.3) | 174 (13.1) | 117 (15.6) | 55 (14.9) | 643 (14.5) |
|  | Total foreigners | - | 465 (46.2) | 537 (39.8) | 460 (34.6) | 253 (33.7) | 131 (35.4) | 1,715 (38.7) |
| Place of contact | | |  |  |  |  |  |  |
|  | supermarket |  | 198 (33.1) | 246 (27.6) | 237 (24.7) | 140 (12.9) | 59 (10.5) | 880 (22.1) |
|  | work |  | 126 (21.0) | 181 (20.3) | 240 (25.0) | 261 (24.0) | 149 (26.5) | 957 (24.0) |
|  | leisure activity |  | 74 (12.4) | 121 (14.3) | 129 (13.5) | 125 (11.5) | 77 (13.7) | 526 (13.2) |
|  | home |  | 70 (11.7) | 121 (13.6) | 127 (13.2) | 215 (19.7) | 84 14.9) | 617 (15.5) |
|  | restaurant/bar | - | - | - | - | 114 (10.5) | 70 (12.5) | 184 (4.6) |
|  | school |  |  |  |  | 36 (3.3) | 26 (4.6) | 62 (1.6) |
|  | other |  | 131 (21.9) | 215 (24.2) | 115 (23.6) | 198 (18.2) | 97 (17.3) | 756 (19.0) |
