# Supplementary material for: Evolving social contact patterns during the COVID-19 crisis in Luxembourg
Source: PLoS One. 2020 Aug 6;15(8):e0237128. doi: 10.1371/journal.pone.0237128 (PMC7410209; doi:10.1371/journal.pone.0237128)
Supplement: S1 File — (PDF) [file pone.0237128.s004.pdf]

Coronavirus crisis: Survey about social distancing in Luxembourg

How well does "social distancing" work in our day-to-day life?

You would like to make a contribution to combat the current coronavirus-crisis? Take part in this survey and help researchers in Luxembourg to find out whether the current restrictive measures are sufficient or not.

Staying at home, keeping your distance in the supermarket, no more access to playgrounds...To what extent have the restrictive measures put in place in Luxembourg to increase physical distancing had an impact on our contact patterns?

The survey is anonymous and takes less than 1 minute to complete. You are asked to indicate your age and nationality, but no other personal data is requested.
The results of the survey will be transmitted to research institutions, statistical offices and ministries in Luxembourg for analysis.

We will do this survey once a week.

Many thanks for your participation!

1. Your age

- ○ 13-17
- ○ 18-24
- ○ 25-34
- ○ 35-44
- ○ 45-54
- ○ 55-64
- ○ 65-74
- ○ 75-84
- ○ 85-94
- ○ 95+

2. Your nationality?

3. With how many other people do you live with in your household?

- 0
- 1
- 2
- 3
- 4
- 5 or more

4. With how many people have you had a personal conversation in the past 24 hours beside the members of your household? This means conversations with more than 3 words, face-to-face, i.e. not via phone or videochat, at a distance below 2m. Members of your own household excluded. Try to think about it carefully and track your daily routine.

- 0
- 1
- 2
- 3
- 4
- 5
- 6-9
- 10 or more

5. If you had contact with people other than the members of your household, where did that happen?

- At work
- At home
- At the supermarket
- During a leisure activity
- Other
