# Supplementary material for: Evolving social contact patterns during the COVID-19 crisis in Luxembourg
Source: PLoS One. 2020 Aug 6;15(8):e0237128. doi: 10.1371/journal.pone.0237128 (PMC7410209; doi:10.1371/journal.pone.0237128)
Supplement: S2 File — (PDF) [file pone.0237128.s005.pdf]

Covid-19 crisis: Survey about social distancing in Luxembourg during lockdown and deconfinement

How well does "social distancing" work in our day-to-day life?

You would like to make a contribution to combat the current coronavirus-crisis? Take part in this survey and help researchers in Luxembourg to find out how our social interactions and contact patterns change during the lockdown and the deconfinement.

The survey is anonymous and takes less than 1 minute to complete. You are asked to indicate your age and nationality, but no other personal data is requested.
The results of the survey will be transmitted to research institutions, statistical offices and ministries in Luxembourg for analysis.

We do this survey regularly, in order to track the change in behaviour during the different phases of the Coronavirs-crisis.

Many thanks for your participation!

1. Your age

- ○ 13-17
- ○ 18-24
- ○ 25-34
- ○ 35-44
- ○ 45-54
- ○ 55-64
- ○ 65-74
- ○ 75-84
- ○ 85-94
- ○ 95+

2. Your nationality?

[ ]

3. With how many other people do you live in your household?

- ○ 0
- ○ 1
- ○ 2
- ○ 3
- ○ 4
- ○ 5 or more

4. With how many people have you had a personal conversation in the past 24 hours beside the members of your household? This means conversations with more than 3 words (with or without mask), face-to-face, i.e. not via phone or videochat, at a distance below 2m. Members of your own household excluded. Try to think about it carefully and track your daily routine.

- ○ 0
- ○ 1
- ○ 2
- ○ 3
- ○ 4

- 5
- 6
- 7
- 8
- 9
- 10
- 11
- 12
- 13
- 14
- 15
- 16
- 17
- 18
- 19
- 20
- 21
- 22
- 23
- 24
- 25 or more

5. Among those people you have had a personal conversation in the last 24 hours (that you indicated in the question before): with how many among them did you have contact without mask?

- 0
- 1
- 2
- 3
- 4
- 5
- 6
- 7

- 8
- 9
- 10
- 11
- 12
- 13
- 14
- 15
- 16
- 17
- 18
- 19
- 20
- 21
- 22
- 23
- 24
- 25 or more

6. If you had contact with people other than the members of your household, where did that happen?

- At work
- At home
- At the supermarket
- At the restaurant
- At a bar
- At school
- During a leisure activity
- Other
